# Editorial: Machine Learning for Water Resources

**DOI:** 10.3389/frai.2021.699862

**Published:** 2021-05-13

**Authors:** Gregoire Mariethoz, J. Jaime Gómez-Hernández

**Affiliations:** ^1^ Institute of Earth Surface Dynamics, University of Lausanne, Lausanne, Switzerland; ^2^ Institute for Water and Environmental Engineering, Universitat Politècnica de València, Valencia, Spain

**Keywords:** water, hydrology, hydrogeology, algorithms, water quality, machine learning, AI

The last years have seen a dramatic increase in the amount of data available to model Earth and environmental systems, thanks to new sensing technologies and open data policies. At the same time, innovative machine learning approaches are being developed, that are ideal tools to extract information from this large amount of data. This conjunction of more data and improved algorithms has a strong impact on research carried out in hydrology and hydrogeology, where non-linear processes are ubiquitous. This is reflected in the papers contained in this Research Topic on Machine Learning for Water Resources. These papers spread a wide range of domains, reflecting the richness in application domains, the wealth of data available, and the diversity of machine learning approaches.

The papers in this Research Topic show the great interest and potential of future developments for artificial intelligence in hydrology. The result is that the contributions are very varied, and we will not attempt to summarize them all here; instead we encourage readers to delve into these papers themselves.

